# Supplementary material for: Proximate Analysis and Nutritional Evaluation of Twenty Canadian Lentils by Principal Component and Cluster Analyses
Source: Foods. 2020 Feb 11;9(2):175. doi: 10.3390/foods9020175 (PMC7073932; doi:10.3390/foods9020175)
Supplement: Supplementary file 1 [file foods-09-00175-s001.pdf]

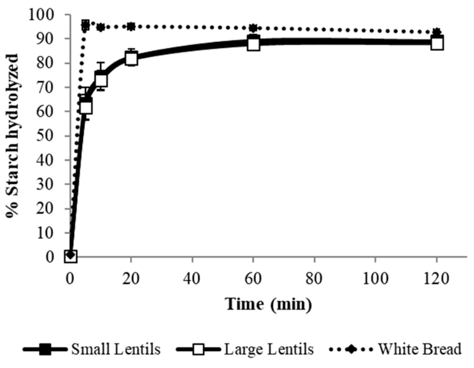

(a) Effect of lentil seed size

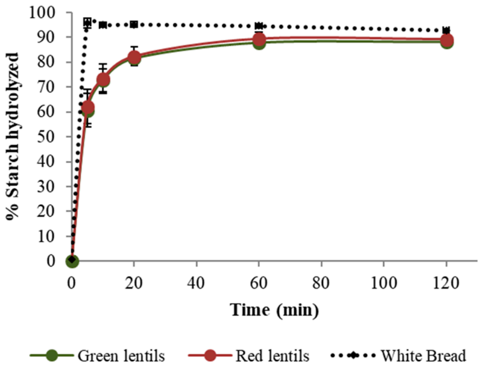

(b) Effect of lentil type

Figure S1. Hydrolysis curves of cooked lentils.
